# Effect of Strengthening Methods on Two-Way Slab under Low-Velocity Impact Loading

**DOI:** 10.3390/ma13245603

**Published:** 2020-12-08

**Authors:** Sun-Jae Yoo, Tian-Feng Yuan, Se-Hee Hong, Young-Soo Yoon

**Affiliations:** School of Civil, Environmental and Architectural Engineering, Korea University, 145 Anam-Ro, Seongbuk-Gu, Seoul 02841, Korea; redtoss@korea.ac.kr (S.-J.Y.); yuantianfeng@korea.ac.kr (T.-F.Y.); bestshhong@korea.ac.kr (S.-H.H.)

**Keywords:** strengthening methods, fiber reinforced polymer, no-slump, high-strength, ductility concrete, low-velocity impact load, two-way slab

## Abstract

In this study, the performance of reinforced concrete slabs strengthened using four methods was investigated under impact loads transferred from the top side to bottom side. The top and bottom sides of test slabs were strengthened by no-slump high-strength, high-ductility concrete (NSHSDC), fiber-reinforced-polymer (FRP) sheet, and sprayed FRP, respectively. The test results indicated that the test specimens strengthened with FRP series showed a 4% increase in reaction force and a decrease in deflection by more than 20% compared to the non-strengthened specimens. However, the specimen enhanced by the NSHSDC jacket at both the top and bottom sides exhibited the highest reaction force and energy dissipation as well as the above measurements because it contains two types of fibers in the NSHSDC. In addition, the weight loss rate was improved by approximately 0.12% for the NSHSDC specimen, which was the lowest among the specimens when measuring the weight before and after the impact load. Therefore, a linear relationship between the top and bottom strengthening of the NSHSDC and the impact resistance was confirmed, concluding that the NSHSDC is effective for impact resistance when the top and bottom sides are strengthened. The results of the analysis of the existing research show that the NSHSDC is considered to have high impact resistance, even though it has lower resistance than the steel fiber reinforced concrete and ultra-high-performance-concrete, it can be expected to further studies on strengthening of NSHSDC.

## 1. Introduction

Concrete structures are likely to be exposed to various impact loadings, for example, rock falls, terrorist attacks, and sudden crashes along their service life. When reinforced concrete slab is subjected to an impact load, it is expected to suffer an important local deflection, secondary damage caused by the occurrence of debris, and internal strength reduction. To prevent such damage, the demand for reinforced concrete with impact resistance has been increasing. Many researchers focus on new concrete members to resist impact loads, such as different reinforcement ratios and materials (containing fibers with various volume fractions, and fiber types) [1,2,3,4]. To improve the impact resistance, developing new construction members is also important, whereas the established concrete members are not only numerous but also less researched. Also, these few research works mainly evaluated the strengthening properties of reinforced concrete (RC) beams. The RC slab strengthened by reinforced materials (FRP and stain hardening cementitious composite) to investigate the impact test is listed in Table 1. They simply evaluated the single strengthening materials (such as just using FRP sheet, without spraying FRP) and strengthened method (without hybrid strengthening). Hence, it is necessary to evaluate the impact resistance in RC slab with a different strengthening method.

The fiber reinforced polymer (FRP) was widely investigated in the RC members for the past few years [16,17,18,19,20,21,22,23,24]. Several researchers have been working toward increasing the concrete strength by attaching carbon fiber sheets (CFS) with high stiffness to the concrete surface [16,17,18]. CFS is not only easy to attach to concrete, but also has a high strength compared to the weight, which makes it attractive. However, the disadvantages of CFS include the requirement of an advanced surface treatment for adhesion with the reinforced concrete, as well the easy CFS peel-off that occurs after a maximum load. Furthermore, an RC member strengthened by CFS can significantly increase bending resistance property, but it does not perform effectively in compression [19].

Also, studies on the strengthening of existing structure through sprayed FRP have recently been conducted [21,22,23,24]. This method is popular because it offers the advantage of easy strengthening compared to the existing retrofitting by FRP. Sprayed FRP is easily implemented by spraying a mix of chopped fibers and resins without being affected by the directionality of the existing FRP. Banathia et al. [21] evaluated reinforced concrete beams through sprayed FRP. The test results indicated that the structure with sprayed FRP had a higher structural performance compared to the non-sprayed reinforced concrete. In addition, Han et al. [23] reinforced a structure using sprayed FRP, which produced isotropic composites; the experimental results indicated excellent structural behavior in the member applying the sprayed FRP. However, strengthening the structure through sprayed FRP may lead to construction errors relative to the design thickness and early detachment due to incomplete hardening of the reinforcing material [24].

Recently, studies on concrete structure that incorporate fibers have been actively conducted [25,26,27,28,29,30,31,32,33]. The fibers employed in ultra-high-performance concrete (UHPC) structure were reported to bring significant effects on the resistance and ductility of the structure after applying maximum loads. However, creating a new structure with UHPC structure is not only economically unviable due to its high cost, but also does not fully serve the purpose of reinforcing existing structure. Therefore, strengthening UHPC in existing concrete structure can be a desirable solution [33,34].

High-performance-fiber-reinforced-cement composites (HPFRCCs) exhibit high strength and are being studied and widely applied [33,34,35,36,37]. Among these HPFRCCs, no-slump high-strength and high-ductility concrete (NSHSDC) offers excellent adhesion with conventional concrete owing to its high viscosity, excellent load resistance, and ductility [35,36]. This is because a hybrid mix of two different fiber lengths is adopted to improve the mechanical properties during the NSHSDC manufacturing [35]. Furthermore, when mixing two types of fibers with different lengths, long fibers are considered to have an outstanding macro-crack control effect, whereas short fibers have excellent microcrack control effects [29]. In addition, NSHSDC confirmed the adhesion to the existing concrete made of high-strength concrete with a strength of 120 MPa [36].

This study evaluated the impact resistance properties for the following four strengthening methods: FRP, sprayed FRP, NSHSDC, and hybrid. Not only the bottom side, but also the top side were strengthened based on the existing research results [33,36]. The hybrid method strengthened the structure with NSHSDC on the top side and sprayed FRP on the bottom side, considering that the sprayed FRP and NSHSDC have excellent tensile and compression strengthening effects, respectively. The crack patterns and failure modes after the experiment are compared and used as reference data for the structural characteristics of reinforced concrete according to the strengthening techniques. Additionally, this study aims to contribute basic data about RC slabs strengthened by different strengthening methods to the resistant impact load.

## 2. Materials and Methods

### 2.1. Mix Proportions and Materials

The following materials were used in the normal concrete (NC) mixture, as shown in Table 2. Portland type 1 cement (specific-surface area 3492 cm^2^/g, density 3.15 g/cm^3^) was used, while river sand was used as the fine aggregate. In addition, crushed stones with a maximum dimension of 20 mm were used for the coarse aggregate. The details are shown in Table 2.

The NSHSDC mixture contains silica fume and silica flour for high strength, resulting in a dense cement matrix. The steel fiber (0.2 mm of diameter, 19.5 mm of length, and 7.8 g/cm^3^ of density) and polyethylene (PE; 31 µm of diameter, 12 mm of length, and 0.97 g/cm^3^ of density) fiber were mixed with volume fractions of 1.0% and 0.5%, respectively, and their physical properties correspond to those of existing studies [35]. Polycarboxylate high-performance reducer (liquid/brown, 1.07 g/cm^3^) is adopted as superplasticizer (SP) at cement weight ratio of 3% [36] to facilitate the mixing of concrete. Table 3 presents the detailed mixture of the NSHSDC. In Table 3, w/b and SF denote the water-to-binder ratio and steel fiber, respectively.

The compressive strength and elastic modulus were obtained by measuring the average compressive strain; the measurement was performed by installing three linear variable differential transformers (LVDT) on a ø100 mm × 200 mm specimen in accordance with ASTM C39 [38]. In addition, the flexural strength was measured in compliance with ASTM C1609 [39] on three specimens of square section with dimensions of 100 mm × 100 mm × 400 mm. The physical properties of NC and NSHSDC are listed in Table 4.

### 2.2. Details of Test Specimens

**Figure 1:**
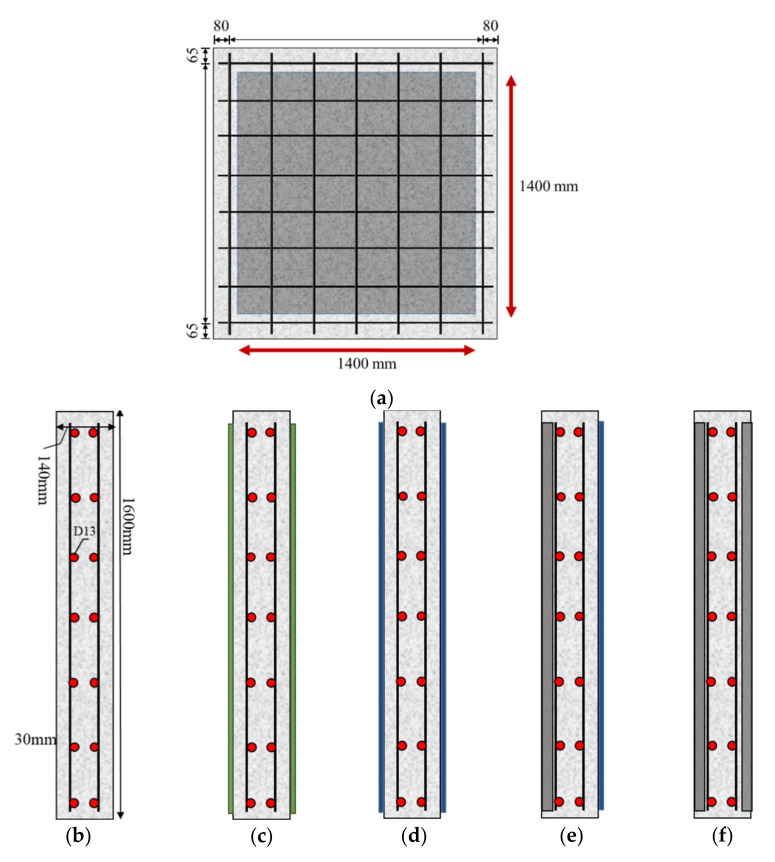
Dimensions and layout of test specimens: (**a**) Dimension of the test specimen; (**b**) NC-NF; (**c**) NC-F; (**d**) NC-F-S; (**e**) NSC-F-S; (**f**) NSC-NF.

**Figure 2:**
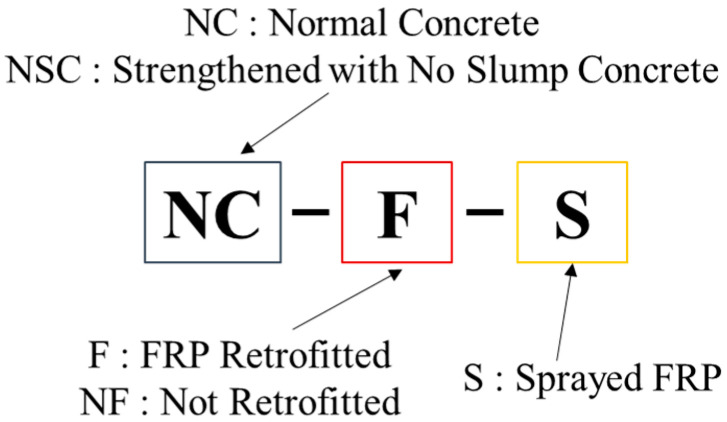
Designation of the test specimens.

A total of six test specimens were tested in which both top and bottom were retrofitted with externally bonded CFS, RC sprayed with carbon fiber roving, strengthened with NSHSDC, and one in which the top was strengthened with NSHSDC and the bottom was sprayed with FRP, as well as one control RC slab and one steel fiber reinforced slab. The concrete specimens are designated as follows: the first string of characters shows the type of concrete used, where NC, NSC, SC denote normal-strength concrete, normal-strength concrete strengthened with NSHSDC, and steel fiber reinforced concrete, respectively; the second string of characters indicates the attachment of FRP; test specimens retrofitted with FRP are denoted by F, and the test specimens without FRP are denoted by NF; and the last string of characters shows that if FRP was sprayed, it was marked as S, and if not, it was not marked. For example, NCS-CF-S designated the specimen for which the bottom side is sprayed with FRP after the top side is strengthened with NSHSDC. Figure 1 and Table 5 present the details of the test specimen. Figure 2 presents the designation of the test specimens.

### 2.3. Strengthening Methods

Figure 1a depicts the shape of the test specimens for examining the impact load. The reinforced concrete slabs present square sections with a width of 1600 mm × 1600 mm and a height of 140 mm. The tensile and compressive reinforcements are achieved by D13 steel bars with diameter of 13 mm. The rebar spacing in the horizontal plane is 240 mm in the x-direction and 210 mm in the y-direction, respectively; the corresponding rebar ratios are 0.38% and 0.43%, respectively. This satisfies the minimum reinforcement criteria specified by ACI 318-19.

CFS was attached in the x-direction of RC slab surface (*ρ*_rebar_ = 0.38%) and applied to the second layer equally in the traversal direction. In this study, the structural strengthening of concrete was conducted using dispensing FRP roving equipment manufactured by Magnum Venus Company (Knoxville, TN, USA) [23]. Carbon fiber roving was used as the experimental material, and vinyl ester was used as resin. The ratio of fiber and resin was set to 2:1 with reference to a previous study [24], and the curing agent was mixed at 2% of the weight of vinyl ester. The length of the carbon fiber strand was set to approximately 40 mm and the thickness was set to 6 mm considering the relationship with the FRP sheet expressed in Equation (1). The properties of the fiber and resin are listed in Table 6.

(1) $$\frac{\sigma_{\mathrm{FRP}}}{\sigma_{\text{Sprayed FRP}}}T_{\mathrm{FRP}}=T_{\text{Sprayed FRP}}$$

where $\sigma_{\mathrm{FRP}}$ is the tensile strength of FRP Sheet, $\sigma_{\text{Sprayed FRP}}$ is the tensile strength of sprayed FRP, $T_{\mathrm{FRP}}$ is the thickness of FRP, and $T_{\text{Sprayed FRP}}$ is the thickness of sprayed FRP.

To overlay the NSHSDC on the specimens, 20 mm of the concrete cover was drilled, which required careful attention because the vibration during the drilling process can deteriorate the durability of the specimens. Subsequently, the NSHSDC was poured into the top and bottom sides of the concrete slab. That is, a structural member in which NSHSDC was strengthened in the compression zone (20 mm) and tensile zone (20 mm). The test specimen obtained by the strengthening method is the same as shown in Figure 1.

### 2.4. Test Setup for Drop-Weight Test

**Figure 3:**
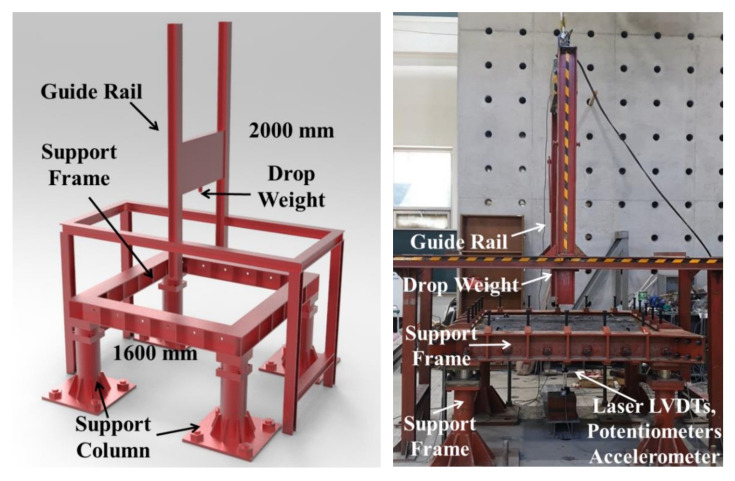
Test setup for drop-weight test.

Figure 3 presents the setup for the drop-weight test of the test specimens. The behavior and maximum load of the test specimens measured during impact loading may exhibit significantly different results depending on the stiffness of the test specimen as well as the load conditions, such as drop weight and impact velocity [37].

As shown in Figure 3, the impact load was applied to the reinforced concrete slab by fitting the weight to the guide, pulling it up using hydraulic pressure, and letting the drop weight of 300 kg freely fall to the center of the slab. To ensure precise impact loading on the test specimens, a guide rail was used to prevent inaccuracy. In addition, a load cell with a capacity of 2000 kN was installed at the top to measure the impact force, and a load cell with a maximum capacity of 500 kN was installed in each of the four supports to measure the reaction force. The impact energy per blow applied to the test specimens was approximately 5.89 kJ at a velocity of 6.3 m/s, and all edges were fixed, with a net span of 1500 mm. The support columns were fixed with steel bars to prevent the uplift of the test specimens. All specimens were subjected to free fall at a height of 2000 mm. The criterion for the end of the experiment was based on the time when the impact or reaction force decreased rapidly, or severe cracks occurred in the specimens.

Instrumentations of the test specimen involve a laser type of LVDT installed at the bottom surface for measuring the deflection. Here, the LVDT is a laser type (Optex, Tokyo, Japan), which has the advantage of being able to record the instantaneous displacement at a rate of hundreds of thousands of data per second. An accelerometer with a limit of 5000 g (g = gravitational acceleration) and potentiometers with a maximum capacity of 100 mm were placed at the bottom of the specimen to measure the displacement. All output data were obtained through a dynamic data logger (DEWE-43, Trbovlje, Slovenia) and real-time impact data was acquired at a frequency of 200 kHz.

## 3. Results and Discussion

### 3.1. Drop-Weight Test

#### 3.1.1. Load-Time Curves

**Figure 4:**
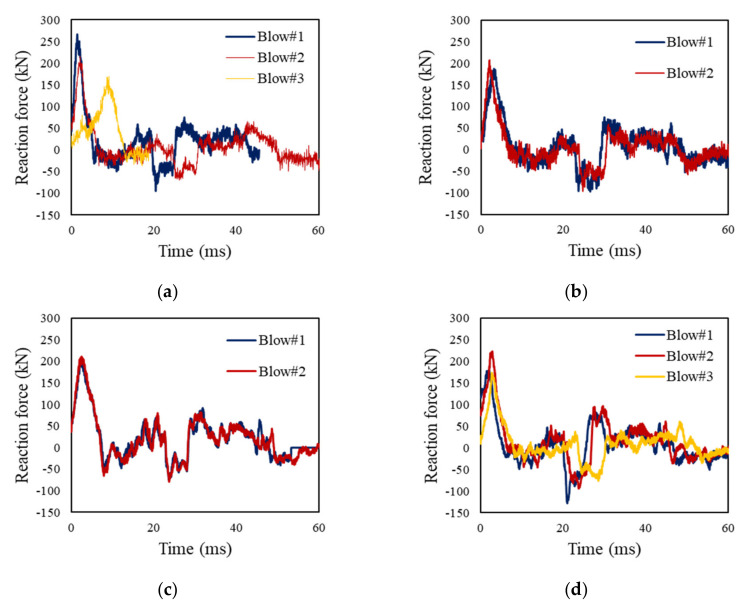
Time history of reaction force at each blow: (**a**) NSC-NF; (**b**) NSC-F-S; (**c**) NC-F-S; (**d**) NC-F.

Figure 4 and Table 7 present the measured reaction force and impact force over time. In comparison with the strengthening techniques, the impact force shows a similar strength at the first blow. This suggests that all initial stiffness values are similar regardless of the type of strengthening method used. As the blows continue, the impact force tends to decrease, which indicates a reduction in the bearing strength of the test specimen. The reaction force represents a load except for loss, such as the inertial force in the impact load, which indicates that the impact force is higher than the reaction force [40,41,42,43]. NSC-NF had a maximum reaction force of approximately 266.63 kN, which is approximately 28% to 43% higher than that of the other strengthened specimens. That is, NSC-NF had a higher strength than the test specimen strengthened with the FRP series at the first blow. This is related to the time delay in the impact energy transfer because of impact load being dominated by inertial force in the early stage, and then the impact force was dissipated, which increased the effect of the reaction force [42,43,44]. At this time, it is determined that the steel fiber and PE fiber in the NSC-series not only generate energy redistribution after the impact load, but also exhibit higher load resistance than the FRP series owing to time delay [45]. In NSC-F-S, the reaction force was measured to be smaller than that of NSC-NF by approximately 22%, but the reaction force was approximately 20% larger than that of NC-F and NC-F-S.

As the blow progressed, the NSC-NF tended to gradually decrease the reaction force as a number of cracks occurred, but the difference between the impact force and the reaction force gradually decreased as the time lag occurred. Accordingly, all the measured reaction forces at the second blows are similar. The reaction force of the other specimens showed a slight increment by approximately 5–20% at the second blow, which increased the reaction force as the impact force applied to the reinforced concrete specimen was transferred to a support column. As shown in Figure 4, the reaction force gradually decreases as the blows progress, and the highest reaction force in NSC-NF is measured.

#### 3.1.2. Deflection-Time Curves

**Figure 5:**
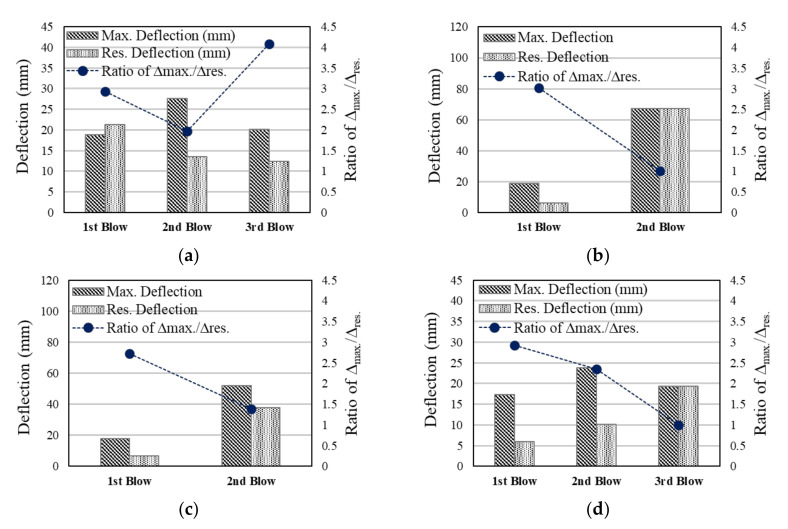
Blow-deflection measurement at each blow under impact loading: (**a**) NSC-NF series; (**b**) NSC-F-S series; (**c**) NC-F-S series; (**d**) NC-F series.

The experimental results are shown in Figure 5 and Table 8. The maximum deflection and residual deflection at the first blow were similar regardless of the strengthening method used. Among these specimens, the maximum deflection and residual deflection generated at the first blow were 17.31 mm and 5.92 mm, respectively, with the test specimen of NC-F showing the least deflection. This enhancement could be credited to the high strength of the CFS in the initial stiffness of the specimen. However, as the blows progressed, the stress concentration on the backside occurred due to accumulated energy, resulting in high maximum and residual deflections. In particular, the test specimen strengthened with sprayed-FRP series showed high maximum deflection and residual deflection at the second blow.

On the other hand, the ratio of ∆_max_/∆_res_ represents the degree of recovery for the structural specimen, where the higher the ratio, the higher the return for deflection [30]. As the blow progresses, this value tends to gradually decrease. The NC-F showed the best ratio of recovery until the second blow owing to the enhancement of the initial stiffness. However, the repeated blow reduced the ratio of recovery at the third blow. In all the test specimens, except for NSC-NF, this ratio was close to 1 at the time of the final blow. Thus, the test specimen attached to the FRP series shows a high degree of recovery and deflection control in the initial blow, but it is deemed necessary to consider the sudden destruction of FRP at the repeated blow. Therefore, the residual ductility ratio, which is the ratio of the maximum deflection and residual deflection, is the lowest. In NSC-NF, the residual ductility ratio is approximately 4 and the ductility ratio is increased at the third blow, which is considered to have not yet reached the ultimate tensile strength due to the crack control effect of the steel fiber and PE fiber [45,46]. However, there was a large deflection in the test specimen, and no further experiment was conducted.

#### 3.1.3. Energy Dissipation Capacity

**Figure 6:**
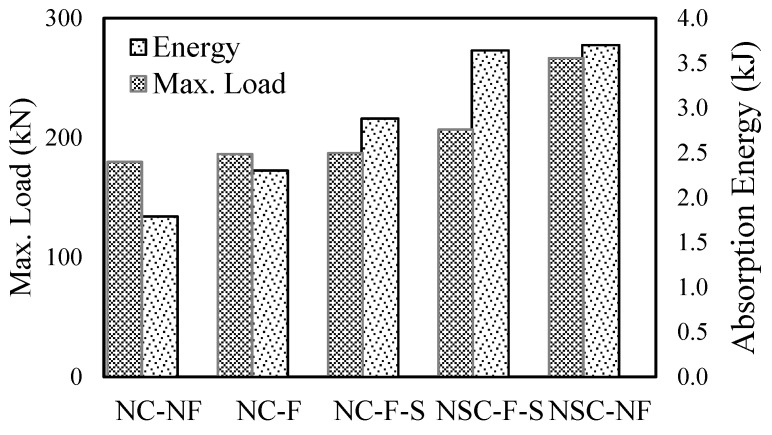
Energy dissipation capacity at first blow.

Figure 6 compares the energy dissipated capacity and maximum reaction force of the test specimens. In other words, the energy dissipated capacity represents the degree of absorption of the impact energy applied to the specimens. The energy dissipation capacity is used as an indicator of the degree of damage to reinforced concrete structure as the impacts drop, but it is less reliable if a large deflection occurs [33]. This was calculated from the section where the maximum deflection occurred in the load–deflection curve to the point where it fell vertically.

In this study, the energy dissipation capacity of a reinforced concrete slab was calculated based on the first blow because the deflection is excessively large at the final blow, resulting in a reliability problem in obtaining the overall energy dissipation capacity [33]. At the first blow, the energy dissipation capacities for all reinforced concrete slabs were approximately 1.79 kJ, 3.70 kJ, 3.64 kJ, 2.3 kJ, and 2.88 kJ, respectively (Figure 6). Overall, the higher the reaction force, the higher the energy dissipation ability. It appears that the energy dissipation capacity increased with the reaction force. The energy dissipation capacity of reinforced concrete slabs strengthened with NSHSDC was higher than that of slabs retrofitted with carbon fibers.

### 3.2. Effect of Carbon Fiber and NSHSDC on Damage Assessment

#### 3.2.1. Damage Assessment Based on Support Rotation

The support rotation angle generated in the test specimen to which the impact load is applied is a criterion for evaluating the residual performance of the structure. According to UFC-3-340-02 [47], one of the commonly used design criteria, the residual performance of the structural specimen is evaluated using the rotation of the support angle. UFC-340-02 [47] defines the degree of damage to the reinforced concrete as light, moderate, or severe according to the generated angle of rotation (Table 9). Although it had three impacts, it was found that the support rotation of the final blow that occurred in the NCS-NF was the least. It appears that the NCS-NF controls the impact load owing to the high rigidity of NSHSDC. In general, the specimen strengthened with sprayed FRP had the highest value of rotation angle compared to the other specimens. The NSC-NF showed the smallest support rotation at the final blow, which is smaller than 2 degrees. It can be expected that NSC-NF is the light level of the damage assessment given in the UFC 3-340-02 criterion [47].

#### 3.2.2. Cracks and Damage

**Figure 7:**
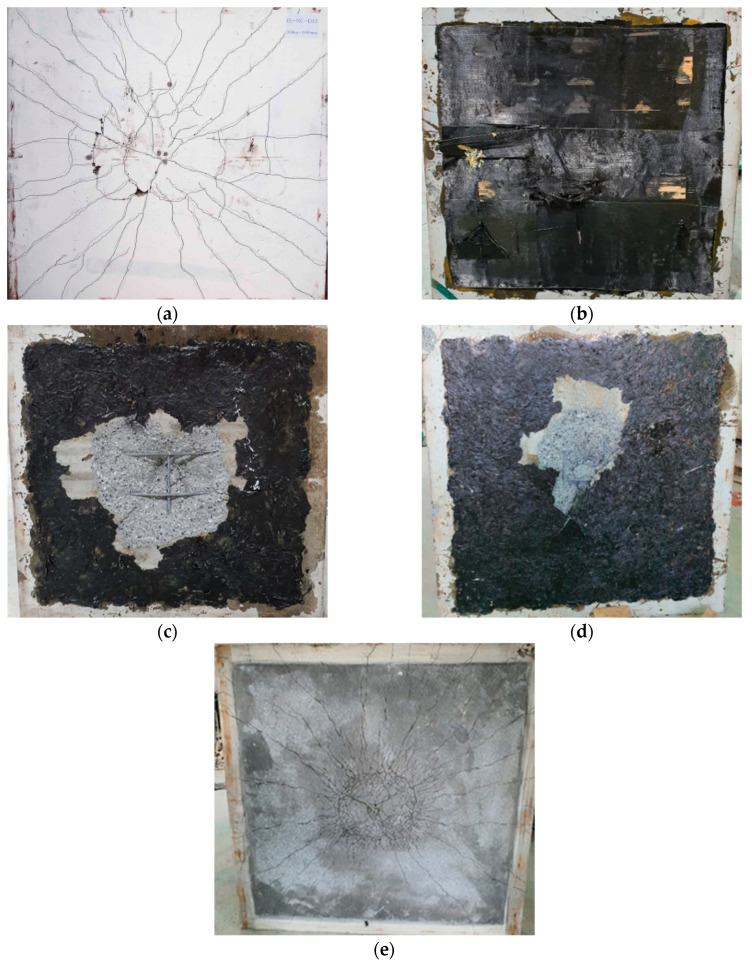
Failure modes of tested specimens of bottom side: (**a**) NC-NF; (**b**) NC-F; (**c**) NC-F-S; (**d**) NSC-F-S; (**e**) NSC-NF.

The pattern of cracks after the experiment is shown in Figure 7. Despite the first blow, the NC-NF had a crack width in the center of the bottom side. The NSC-NF showed several bending cracks, most of which were identified near the central part of the it because of the bridging effect of the steel and PE fibers [36,37]. A lot of cracks were found on the back side, and the damaged area was the smallest. Although it is not a comparison for the same blow, it is found that the micro cracks are much more frequent than the NC-NF.

On the other hand, the test specimen strengthened with the sprayed FRP (NC-F-S) was found to have significant damage to the back side. The tensile reinforcement was exposed, and the damaged area was found to be wider than that of the other test specimens owing to the characteristic carbon fiber roving; although the test specimen (NC-F-S) had a high resistance to the impact energy due to the high stiffness of the reinforcement at the first blow, the accumulated impact energy during the repeated blows was determined to exceed the tensile limit of the concrete [46]. In the event of an impact loading, unlike the static load condition, a large local energy was transmitted to the component, which was reinforced with sprayed FRP, and a large stress was concentrated on the backside, resulting in major destruction at the second blows [40]. Therefore, the NC-F subjected to impact loading is thought to have higher strength than that of NC-F-S. Meanwhile, hardly any cracks were identified on the NC-F due to the attachment of the carbon fiber; however, at the time of final destruction, the delamination of CFS occurred. The strength of the interface attachment between the concrete and the CFS due to the repeated blows was reduced owing to the impact load. Therefore, the least amount of damage can be expected when the top and bottom parts are strengthened with NSHSDC.

#### 3.2.3. Weight Loss

**Figure 8:**
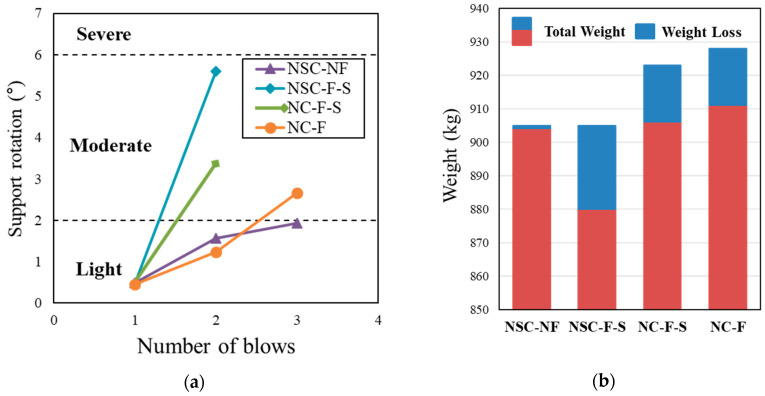
Support rotation and weight loss by impact loading: (**a**) Support rotation; (**b**) Weight loss.

The weight loss rate is an additional indicator of the residual performance after an impact load. The occurrence of concrete debris due to an impact load not only reduces the usability of the structure, but also causes environmental damage. Several studies are currently being conducted to reduce debris in specimens [13,48]. In this study, the residual structural performance of concrete slabs under an impact load was evaluated by comparing the occurrence rate of fragments, as shown in Figure 8. All specimens, including the control slab, did not exhibit any loss of weight on the first blow. Further experiments were not conducted for the NC-NF because they were fabricated as a comparison data to determine the strengthening effects of the test specimens. NSC-F-S and NC-CF-S showed the highest weight loss rate of approximately 5%. The NSC-NF showed a weight loss of only 1 kg, which was significantly small (approximately 0.12% of the total slab weight). This indicates that the weight loss rate of the test specimen strengthened with FRP on the bottom side is approximately 3–5%, and the lowest weight loss rate can be expected when strengthening both top and bottom sides with NSHSDC jacket. Considering the damaged areas, it is similar regardless of the test specimens. The result suggests that the brittle fracture on the top side can be fully controlled as the strengthening progresses. However, it is expected to be the least damaged in the test specimen strengthened with the NSHSDC, and the largest damaged area of failure was shown in the test specimen strengthened with the sprayed-FRP series.

### 3.3. Comparison of Previous and Experimental Test

This study evaluated the effects of reinforced concrete according to various strengthening methods. The test results showed the best impact resistance properties were shown in the NSC-NF. Furthermore, this study analyzed the two-way slabs with a similar impact energy of existing studies, as shown in Table 10. The comparison slab of the analysis consists of specimens strengthened with FRP, steel fiber reinforced concrete (SFRC), and UHPC. Bhatti et al. [6] studied the test specimens retrofitted by FRP sheets, and the size of the slab is similar to the specimen performed in this study. The reaction force was slightly smaller than that of NSC-NF, but there was a large deflection and residual deflection compared to the specimen used in this study. This suggests that the low strength of the FRP sheet is less effective than strengthening materials used in this paper. The approximate energy dissipated capacity was somewhat larger than this study, which is more exacerbated by the fact that the high rebar ratio was used, and the size of the slab was large. Trevor et al. [4] performed the drop-weight test with SFRC. The reaction force of the slab was higher than the results of this study, which is considered to have improved impact resistance due to the high rebar ratio and bridging effect of the steel fibers. Jang [49] implemented the impact test under the same conditions as the test specimens and 1% of the steel fibers were mixed with volume fractions of 1.0%. The maximum deflection was larger than that of NSHSDC in this study because the layer of NSHSDC controlled the deflection. However, the reaction force and energy dissipated ability of the SFRC is about 34% and 24% higher than NSC-NF, respectively. Although the impact resistance of NSC-NF is somewhat lower than that of the SFRC, it can be judged that the strengthening efficiency is high in that the thickness of NSHSDC is less than 30% of the thickness of SFRC. Kim [50] produced UHPC with a thickness of 25% thinner than the test specimen and the impact test was performed. The results of the experiment showed similar reaction force and deflection with NSC-NF, but the thickness of NSHSDC is only 38% of UHPC.

## 4. Conclusions

This study evaluated the effects of reinforced concrete according to various reinforcement methods. Five test specimens were fabricated, including control specimens, and three strengthening methods were applied. The following results were derived from the experimental results:

(1) The NSC-NF showed excellent impact resistance and high strength. In particular, the highest strength and lowest deflection were obtained when strengthening the top and bottom sides with NSHSDC; the cracks were confirmed to spread evenly under the test specimens. This is because steel fibers and PE fibers included in the NSHSDC effectively control macro-cracking and micro-cracking and spread impact energy evenly throughout the test specimen.
(2) Comparing deflection at the first blow, the lowest maximum deflection was observed in the NC-F specimen, which was 26% lower than that of the control specimen. This is attributed to the fact that CFS increased the initial stiffness. Overall, at the first blow, the carbon fiber series showed less deflection than the NSHSDC series; however, at the final blow, the ratio of maximum deflection to residual deflection decreased.
(3) The crack of NSC-NF not only spread the crack evenly at the bottom but also improved the deflection capacity according to using NSHSDC, which was a hybrid using PE and steel fibers. However, strengthening with FRP and sprayed FRP cannot confirm the cracks due to the FRP adhesion. The NC-F was delaminated with the FRP, which showed the largest damaged area and exposed the tensile reinforcement. The sprayed FRP may have caused brittle fracture due to the stress concentration at the tensile section.
(4) The NSC-NF showed a weight loss ratio of about 1 kg on the final blow, which is only about 0.12% of the total weight. However, the weight loss rate of the test specimen strengthened with FRP on the bottom side is approximately 2–3% of the total weight. This is due to the high ductility of the specimen as a bridging effect of steel fiber and PE fiber. Thus, the measured weight loss of the NSC-NF was smaller than that of the FRP series.
(5) Compared with the previous studies, NSC-NF showed best impact resistance in the test specimens applying the strengthening techniques, but it showed disadvantage in load and deflection SFRC concrete and UHPC. However, it can be expected to further research studies on the applicability of the NSHSDC in that it can be manufactured with the thin layer while strengthening the existing concrete.

## Figures and Tables

**Table 1:** Recent research on reinforced concrete (RC slabs strengthened by different materials).

Researchers			Strengthening Materials	Evaluation Method
1.	Foret et al. 2008	[5]	CFRP strip (two types spacing)	Numerical analysis
2.	Bhatti et al. 2011	[6]	FRP sheet (AFRP and CFRP sheet)	Test
3.	Yoo et al. 2014	[7]	FRP sheet (AFRP and CFRP sheet)	Test and numerical analysis
4.	Radnic et al. 2015	[8]	FRP strips (two types strengthening method)	Test and numerical analysis
5.	Wang et al. 2018	[9]	Flax FRP (different strengthening method)	Test
6.	Yilmaz et al. 2018	[10]	CFRP strips (two types strengthening method)	Test and numerical analysis
7.	Elnagar et al. 2019	[11]	Strain-hardening cementitious composites	Test and numerical analysis
8.	Yang et al. 2019	[12]	FRP sheet (different strengthening method)	Numerical analysis
9.	Soltani et al. 2020	[13]	GFRP sheets (different strengthening method)	Test and numerical analysis
10.	Mugunthan et al. 2020	[14]	GFRP strips (different strengthening method)	Test
11.	Mahmoud et al. 2020	[15]	Strain-hardening cementitious composites	Test

**Table 2:** Mix proportions of concrete (by cement weight ratio).

Type	w/b (%)	Unit Weight			
		Water	Cement	Fine Aggregate	Coarse Aggregate
NC	33	0.33	1.00	1.43	1.70

NC = normal strength concrete.

**Table 3:** Mix proportions of no-slump high-strength, high-ductility concrete (NSHSDC; by cement weight ratio).

Type	w/b (%)	Unit Weight					Fiber		SP
		Water	Cement	Silica Fume	Silica Filer	Silica Sand	SF	PE	
							(%)		
NSHSDC	17.2	0.215	1.00	0.25	0.30	1.10	1.0	0.5	3.0%

NSHSDC = no-slump high-strength, high-ductility concrete; w/b = water to binder ratio; SF = steel fiber; PE = polyethylene fiber; SP = superplasticizer.

**Table 4:** Mechanical properties of concrete.

Specimens	Compressive Strength (MPa)	Flexural Strength (MPa)	Elastic Modulus (GPa)
NC	40.7	4.24	27.42
NSHSDC	123.9	18.52	41.26

**Table 5:** Designation and specifications of the test specimens.

Designation	Type of Strengthening Methods			Strengthening Thickness (mm)	
	NSC	Sprayed FRP	FRP Sheet	Top	Bottom
NC-NF	-	-	-	-	-
NC-F	-	-	○	0.34	0.34
NC-F-S	-	○	-	6	6
NSC-F-S	○	○	-	20	6
NSC-NF	○	-	-	20	20

NSC = no-slump high-strength, high-ductility concrete.

**Table 6:** Mechanical properties of carbon fiber and resin.

Series	Carbon Fiber		Resin	
	Sheet	Roving	Epoxy	Vinyl Ester
Tensile strength (MPa)	4900	4200	90	88
Elastic modulus (GPa)	230	240	30	7.3
Ultimate strain (%)	2.1	1.8	8.0	4.5
Thickness (mm)	0.167	3	-	-

**Table 7:** Results of impact test of two-way slabs.

Test Members	Blow No.	Impact Properties			Max. Midspan Displacement	Residual. Midspan Displacement	Max. Reaction Force
		H (mm)	E_i_ (kJ)	F_i_ (kN)	D_max_ (mm)	D_res_ (mm)	F_r_ (kN)
NC-NF	1	2000	5.89	410.10	23.41	7.75	179.75
NC-F	1	2000	5.89	611.97	17.31	5.92	186.36
	2			320.96	23.80	10.15	225.52
	3			282.16	19.33	19.29	176.20
NC-F-S	1	2000	5.89	637.58	17.91	6.57	187.16
	2			369.29	51.96	37.60	212.64
NSC-F-S	1	2000	5.89	587.50	18.89	6.24	206.84
	2			298.40	67.29	67.24	217.14
NSC-NF	1	2000	5.89	636.88	18.77	6.39	266.63
	2			450.13	27.65	14.07	216.97
	3			281.52	20.12	4.92	170.35

**Table 8:** Displacements of strengthened concrete slabs under impact loading.

Test Members	Blow No.	Mid-Span Displacement		
		Max. Displacement (mm)	Residual Displacement (mm)	Ratio of ∆_*max*_/∆_*res*_
NC-NF	1	23.47	7.75	3.021
NC-F	1	17.31	5.92	2.924
	2	23.80	10.15	2.345
	3	19.33	19.29	1.002
NC-F-S	1	17.91	6.57	2.726
	2	51.96	37.6	1.382
NSC-F-S	1	18.89	6.24	3.027
	2	67.29	67.24	1.001
NSC-NF	1	18.77	6.39	2.937
	2	27.65	14.07	1.965
	3	20.12	4.92	4.089

**Table 9:** Criteria of damage level according to UFC 3-340-02 [47].

Damage Level	Light	Moderate	Severe
Support rotation criteria	0° ≤ θ ≤ 2°	2° < θ ≤ 6°	6° < θ ≤ 8°

**Table 10:** Recent research about two-way RC slab under impact loadings.

No.	Researcher		Test Specimens	Impact Energy (kJ)	Reaction Force (kN)	Max. Deflection (mm)	Dissipated Energy (kJ)
1.	This study		NC-NF	5.89	179.75	23.41	1.79
2.	This study		NC-F	5.89	186.36	17.31	2.3
3.	This study		NC-F-S	5.89	187.16	17.91	2.88
4.	This study		NSC-NF	5.89	266.63	18.77	3.7
5	This study		NSC-F-S	5.89	206.84	18.99	3.64
6.	Hrynyk et al. 2014	[4]	Steel fiber (1%)	4.8	591	14	3.5
7.	Hrynyk et al. 2014	[4]	Steel fiber (2%)	4.8	621	13.5	4.2
8.	Hrynyk et al. 2014	[4]	Steel fiber (3%)	4.8	531	12.4	3.3
9.	Bhatti et al. 2011	[6]	CFRP strips	5.4	240	32	3.84
10.	Amira et al. 2019	[11]	Cementitious composites	5.93	61	2.65	2.51
11.	Mugunthan et al. 2020.	[14]	GFRP strips	4.86	-	-	-
12.	Jang 2015	[49]	Steel fiber (1%)	5.89	408.6	20.08	4.85
13.	Kim 2017	[50]	UHPC	6.13	272.72	17	4.64
